# Attenuation of cognitive impairment by the nonbacteriolytic antibiotic daptomycin in Wistar rats submitted to pneumococcal meningitis

**DOI:** 10.1186/1471-2202-14-42

**Published:** 2013-04-02

**Authors:** Tatiana Barichello, João Carlos Nepomuceno Gonçalves, Jaqueline S Generoso, Graziele L Milioli, Cintia Silvestre, Caroline S Costa, Jaqueline da Rosa Coelho, Clarissa M Comim, João Quevedo

**Affiliations:** 1 Laboratório de Microbiologia Experimental, Programa de Pós-Graduação em Ciências da Saúde, Unidade Acadêmica de Ciências da Saúde, Universidade do Extremo Sul Catarinense, Criciúma, SC, 888806-000, Brazil; 2 Laboratório de Neurociências, Programa de Pós-Graduação em Ciências da Saúde, Unidade Acadêmica de Ciências da Saúde, Universidade do Extremo Sul Catarinense, Criciúma, SC, 888806-000, Brazil; 3 Instituto Nacional de Ciência e Tecnologia Translacional em Medicina (INCT-TM), Programa de Pós-Graduação em Ciências da Saúde, Unidade Acadêmica de Ciências da Saúde, Universidade do Extremo Sul Catarinense, Criciúma, SC, 88806-000, Brazil; 4 Núcleo de Excelência em Neurociências Aplicadas de Santa Catarina (NENASC), Programa de Pós-Graduação em Ciências da Saúde, Unidade Acadêmica de Ciências da Saúde, Universidade do Extremo Sul Catarinense, Criciúma, SC, 88806-000, Brazil

**Keywords:** *Streptococcus pneumoniae*, Meningitis, Behavior, Memory, Daptomycin

## Abstract

**Background:**

*Streptococcus pneumoniae* is associated with neurologic sequels, such as, seizures, sensory-motor deficits, hearing loss, learning and memory impairment, which can occur in approximately 30 to 52% of surviving patients. Neuronal damage can be caused by intense inflammatory reaction and direct effects of the bacteria virulence factors. The aim of the present study was to evaluate the effects of the nonbacteriolytic antibiotic daptomycin versus ceftriaxone on behavioral parameters in adult Wistar rats submitted to pneumococcal meningitis.

**Results:**

Ten days after induction we verified that the meningitis group with daptomycin treatment showed retention of aversive memory; it presented memory of the object recognition at short term and long term. In continuous multiple-trials step-down inhibitory avoidance task the meningitis group with ceftriaxone treatment required approximately two times more stimulus to reach the acquisition criterion when compared with meningitis group with daptomycin treatment. However, in the habituation memory test there were no differences in the number of crossings and rearings in training and task sessions demonstrating habituation impairment to the environment task in both meningitis groups.

**Conclusions:**

The evidence of the present study shows the potential alternative of the treatment with daptomycin in preventing learning and memory impairments caused by pneumococcal meningitis. Further investigations are necessary to provide support for evaluation of daptomycin as an alternative treatment of bacterial meningitis.

## Background

Pneumococcal meningitis is a life-threatening disease associated with high mortality and morbidity rates. *Streptococcus pneumoniae* meningitis mortality ranges from 16 to 37% and it is associated with neurologic sequels, such as, seizures, sensory-motor deficits, hearing loss, learning and memory impairment, which can occur in approximately 30 to 52% of surviving patients [1-5].

This microorganism can multiply within the cerebrospinal fluid (CSF) and it leads to the release of bacterial components, which stimulates the production of cytokines and other pro-inflammatory molecules in response to bacterial stimuli [6]. As consequence, polymorphonuclear are attracted, activated and released in large amounts of superoxide anion and nitric oxide, leading to oxidative stress. This cascade leads to mitochondrial damage and blood–brain barrier breakdown. Both damages contribute to cell injury during pneumococcal meningitis. The immune response in cerebrospinal fluid has shown to play a key role in this pathophysiology, principally to the development of the brain damage [7]. Neuronal damage can be caused by intense inflammatory reaction and by direct effects of the bacteria virulence factors [8]. The hippocampus is surrounded by interstitial fluid which is contiguous with the CSF, allowing bacterial toxins and pro-inflammatory mediators to propagate into the parenchyma [9].

A nonbacteriolytic antibiotic but with high bactericidal properties would minimize the cognitive damage. Since, during the treatment with bacteriolytic antibiotics it could contribute to increase the inflammation in the subarachnoid space also through the release of bacterial components [10]. Daptomicyn is an antibacterial agent active against Gram-positive bacteria [11]. The bactericidal activity occurs by irreversible binding within the bacterial cell membrane in a calcium-dependent process. This leads to despolarization of the cell membrane and inhibition of the RNA, DNA and protein synthesis, which results in rapid bacterial cell death without triggering immediate cell lysis [12]. Thus, in experimental pneumococcal meningitis it has been demonstrated that daptomicyn produces an enhanced bactericidal activity [13]; it attenuates the CSF inflammation [14] and also prevented cortical brain injury when compared to ceftriaxone treatment [15].

In this context, the aim of the present study was to evaluate the effects of the nonbacteriolytic antibiotic daptomycin versus ceftriaxone on behavioral parameters in adult Wistar rats submitted to pneumococcal meningitis.

## Methods

### Infecting organism

*S*. *pneumoniae* (serotype 3) was cultured overnight in 10 ml of Todd Hewitt broth, diluted in fresh medium and grown to logarithmic phase. This culture was centrifuged for 10 min at (5,000×g) and resuspended in sterile saline to the concentration of 5×10^9^ cfu/ml. The size of the inoculum was confirmed by quantitative cultures [16,17].

### Animal model of meningitis

Adult male Wistar rats (250–350 g body weight), from our breeding colony were used for the experiments. All procedures were approved by the Animal Care and Experimentation Committee of UNESC, Brazil, and followed in accordance with the National Institute of Health Guide for the Care and Use of Laboratory Animals (NIH Publications No. 80–23) revised in 1996. All surgical procedures and bacterial inoculations were performed under anesthesia, consisting of an intraperitoneal administration of ketamine (6.6 mg/kg), xylazine (0.3 mg/kg), and acepromazine (0.16 mg/kg) [18]. Rats underwent a cisterna magna tap with a 23-gauge needle. The animals received either 10 μl of sterile saline as a placebo or an equivalent volume of *S*. *pneumoniae* suspension. At the time of inoculation, animals received fluid replacement and were subsequently returned to their cages [19,20]. Eighteen hours later, the meningitis was documented by a quantitative culture of 5 μl of CSF obtained by puncture of the cisterna magna [16]. The animals were randomly in three different groups sham (control group), meningitis with daptomicyn treatment and meningitis with ceftriaxone treatment. The daptomycin (Cubicin®; 50 mg/kg body weight) and ceftriaxone (100 mg/kg body weight) were administered subcutaneously [s.c.]) during 7 days.

After 10 days, the animals were free from infection. We performed blood cultures that were all negative in this period. Thus, the animals were separately undergone to four behavioral tasks: a) open field; b) object recognition; c) step-down inhibitory avoidance task (single-training) and d) continuous multiple-trials step-down inhibitory avoidance task.

### Behavioral tasks

The animals underwent separately to four behavioral tasks: habituation to an open field, step-down inhibitory avoidance task, continuous multiple-trials step-down inhibitory avoidance task and object recognition. All behavioral procedures were conducted between 13:00 and 16:00 p.m. in a sound-isolated room, and each animal performed only one behavior test. All behavioral tests were recorded by the same person who was blind to the animal group.

### Open field test

The behavior was assessed in the open field apparatus in order to evaluate both locomotor and exploratory activities. The apparatus is a 40 cm × 60 cm open field surrounded by 50 cm high walls made of brown plywood with a frontal glasswall. The floor of the open field is divided into 9 rectangles by black lines. The animals were gently placed on the left rear quadrant and then left alone to explore the arena for 5 min (training session). Immediately after this procedure, the animals were taken back to their home cage and 24 h later they were submitted again to a similar open-field session (test session). Every cross of the black lines and rearing performed in both sessions were counted for 5 min. The decrease in the number of crossings and rearings between the two sessions was taken as a measure of the retention of habituation memory [21].

### Object recognition

This task evaluates the non-aversive and non-spatial memory. The apparatus and procedures for the object recognition task have been described elsewhere [22]. Briefly, the task took place in a 40 × 50 cm open field surrounded by 50 cm high walls made of plywood with a frontal glass wall. The floor of the open field was divided into 12 equal rectangles by black lines. All animals were submitted to a habituation session where they were allowed to freely explore the open field for 5 min. No objects were placed in the box during the habituation trial. The total number of crossings of the black lines and rearings performed in this session were evaluated as locomotors and exploratory activity, respectively. The training was conducted by placing individual rats for 5 min in the field, in which two identical objects (objects A1 and A2, both being cubes) were positioned in two adjacent corners, 10 cm from the walls. In a short-term recognition memory test given 1.5 h after training, the rats explored the open field for 5 min in the presence of one familiar (A) and one novel (B, a pyramid with a square-shaped base) object. All objects had similar textures (smooth), colors (blue), and sizes (weight 150–200 g), but distinctive shapes. A recognition index calculated for each animal is reported as the ratio TB/(TA + TB) (TA = time spent exploring the familiar object A; TB = time spent exploring the novel object B). In a long-term recognition memory test given 24 h after training, the same rats were allowed to explore the field for 5 min in the presence of the familiar object A and a novel object C (a sphere with a square shaped base). Recognition memory was evaluated using the same method of the short-term memory test. Exploration was defined as sniffing (exploring the object 3–5 cm away from it) or touching the object with the nose and/or forepaws.

### Step-down inhibitory avoidance task

This task evaluates aversive memory. The apparatus and procedures have been described in previous reports [23,24]. Briefly, the training apparatus was a 50 × 25 × 25 cm acrylic box (Albarsch, Porto Alegre, Brazil) which the floor was consisted of parallel caliber stainless steel bars (1 mm diameter) spaced 1 cm apart from each other. A 7 cm-wide, 2.5 cm-high platform was placed on the floor of the box against the left wall. In the training trial, animals were placed on the platform and their latency to step down on the grid with all four paws was measured with an automatic device. Immediately after stepping down on the grid, the animals received a 0.4 mA, 2.0 s foot shock and returned to their home cage. A retention test trial was performed 24 h after training (long-term memory). The retention test trial was procedurally identical to the training, except that no foot shock was presented. The retention test step-down latency (maximum, 180 s) was used as a measure of inhibitory avoidance retention. Reactivity to the foot shock was evaluated in the same apparatus used for inhibitory avoidance, except that the platform was removed. Each animal was placed on the grid and allowed 1 min for habituation period prior to the start of a series of shocks (0.5 s) delivered at 10 s intervals. The shock intensities ranged from 0.1 to 0.5 mA in 0.1 mA increments. The adjustments in the shock intensity were made in accordance to each animal’s response. The intensity was raised by 1 unit when no response occurred and lowered by 1 unit when a response was made. A “flinch” response was defined as the withdrawal of one paw from the grid floor, and a “jump” response was defined as rapid withdrawal of three or four paws. Two measurements of the “flinch” threshold were made and then two measurements of the “jump” threshold were made. For each animal the mean of the two scores for the flinch and the jump thresholds were calculated [25,26].

### Continuous multiple-trials step-down inhibitory avoidance task

This task evaluates aversive memory in the test section and learning when analyzing the number of training trials required for the acquisition criterion (see below). It was performed in the same step-down inhibitory avoidance apparatus; however, in the training session, the animal was placed on the platform and immediately after stepping down on the grid, received a 0.3 mA, 2.0 s foot shock. This procedure continued until the rat remained on the platform for 50 s. The animal was then returned to the home cage. The number of training trials required to reach the acquisition criterion of 50 s on the platform was recorded. The retention test was performed 24 h later (long-term memory) [27].

### Statistics

Data from the habituation to an open field task is reported as mean ± SEM, and it was analyzed by the paired Student’s *t* test and ANOVA post-hoc Tukey. Data from the object recognition task and inhibitory avoidance task are reported as median and interquartile ranges, and comparisons among groups were performed using Mann–Whitney U tests. The within-individual groups were analyzed by Wilcoxon’s tests. Data from continuous multiple-trials step-down inhibitory avoidance task the trials were reported as mean ± SD, and were analyzed by the ANOVA post-hoc Tukey. Data from the latency time was reported as median and interquartile ranges, and comparisons among groups were performed using Mann–Whitney U tests. In all comparisons, p<0.05 indicated statistical significance.

## Results

**Figure 1:**
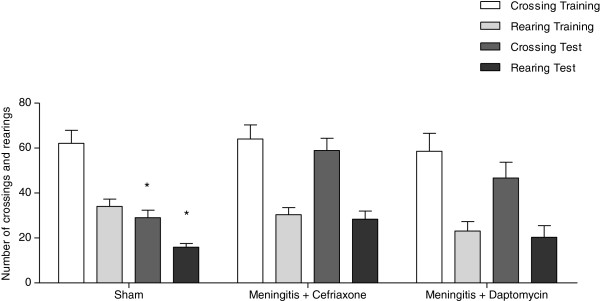
**Open field test 10 days after induction of meningitis by *S. pneumoniae. ***Data are reported as mean ± SEM. n=10 per group, and were analyzed by the paired Student’s *t* test and ANOVA post-hoc Tukey. *p<0.05 vs. Training.

In the open-field task, Figure 1, there were no differences in the number of crossings and rearings among groups in the habituation to the open-field training session (p>0.05) demonstrating no difference in motor and exploratory activity among groups. In the sham group, there was statistical difference between training and test session (crossing t = 9.161, df = 9, p = 0.0001; rearings t = 10.672, df = 9, p = 0.0001) demonstrating habituation memory.

In the meningitis/ceftriaxone group (crossing t = 1.929, df = 9, p = 0.086; rearings t = 0.949, df = 9, p = 0.369) and the meningitis/daptomycin group (crossing t = 2.195, df = 9, p = 0.056; rearings t = 0.884 df = 9, p = 0.400) there were no differences between training session and test session suggesting memory impairment in both groups.

**Figure 2:**
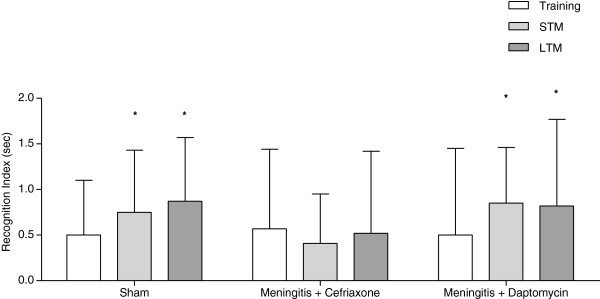
**Object recognition task 10 days after induction of meningitis by *S. pneumoniae. ***The short-term recognition memory the test is evaluated at 1.5 h after training (STM) and the long-term recognition memory the test is evaluated at 24 h after training (LTM). Data are reported as median and interquartile ranges, and comparisons among groups were performed using Mann–Whitney U tests, n=10 per group. The within-individual groups were analyzed by Wilcoxon’s tests. *p<0.05 test vs. training.

The object recognition task in Figure 2, the animals of meningitis/cefriaxone group presented impairment of novel object recognition memory, i.e., they did not spend a significantly greater time exploring the novel object, presenting memory impairment during short term (Z =−0.866, p = 0.386) and long term memory (Z = −0.051, p = 0.959). However, the animals of meningitis/daptomycin group did not present memory impairments during short term (Z = −2.499, p = 0.012) and long term (Z = −2.395, p = 0.017) retention test sessions in comparison to the training trial.

**Figure 3:**
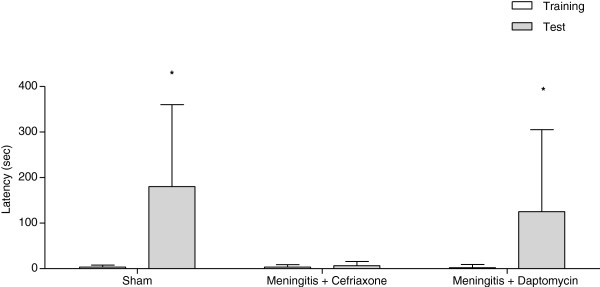
**Latency to step**-**down in the inhibitory avoidance task 10 days after induction of meningitis by *S.pneumoniae. ***Data are reported as median and interquartile ranges, and comparisons among groups were performed using Mann–Whitney U tests, n=10 per group. The within-individual groups were analyzed by Wilcoxon’s tests. *p<0.05 vs. training.

Figure 3, the step-down latency. In the training session there was not significant difference in the latency time among the groups (p>0.05). In the meningitis/ceftriaxone group there wasn’t difference in the latency time between training and test (Z=−1.703; p=0.089) presenting memory impairment. In the meningitis/daptomycin group there was difference between training and test session (Z=−2.810; p=0.005) demonstrating aversive memory in this group.

**Figure 4:**
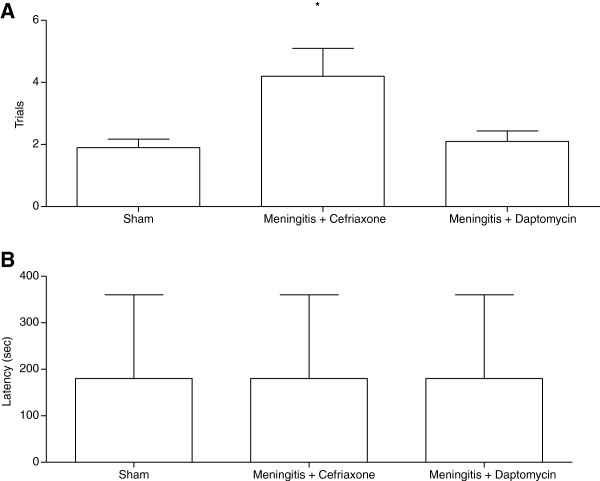
**Continuous multiple trials step**-**down inhibitory avoidance task 10 days after induction of meningitis by *S. pneumoniae. ***Data from the trials is reported as mean ± SD, and were analyzed by the ANOVA post-hoc Tukey, n=10 per group, Figure 4**A**. *p<0.05 vs. Sham. Data from the latency time is reported as median and interquartile ranges, and comparisons among groups were performed by Wilcoxon’s tests, Figure 4**B**. *p<0.05 vs. Sham.

Figure 4, continuous multiple trials step-down inhibitory avoidance. We demonstrated a significant increase in the number of training trials (t = 3.536, df = 18.541, p = 0.002) required to reach the acquisition criterion (50 s on the platform) in the meningitis/ceftriaxone group when compared to the sham group, Figure 4A. The results of this task suggest that the meningitis/ceftriaxone group required approximately two times more stimulus to reach the acquisition criterion when compared with the sham group and with meningitis/daptomycin group. Meningitis/ceftriaxone group had learning and impairment memory. In the retention test, there was no difference among groups for all the times tested, Figure 4B.

## Discussion

In spite of significant advances in pneumococcal meningitis treatment, it remains one of the most important worldwide infectious diseases and it is still correlated with elevated mortality and morbidity. Moreover, a large number of survivors present permanent neurological sequelae [5,6].

The present study suggests a beneficial effect of therapy with daptomycin on memory and learning impairments in an animal model of pneumococcal meningitis. In the habituation memory test there was no difference between treatments with daptomycin and ceftriaxone, both groups demonstrated impairments in this memory. However, the meningitis group with daptomycin treatment showed retention of aversive memory and the animals also presented memory of the object recognition at short term and long term. In continuous multiple-trials step-down inhibitory avoidance task, the meningitis group with ceftriaxone treatment required approximately two times more stimulus to reach the acquisition criterion when compared with meningitis group with daptomycin treatment.

Several studies with daptomycin treatment on experimental pneumococcal meningitis with favorable results have been reported. Daptomycin cleared the bacteria more efficiently from the CSF than ceftriaxone; decreased the inflammatory host response, as assessed by the matrix metalloproteinase-9, IL-1β, IL-10, IL-18, monocyte chemoattractant protein 1 (MCP-1), macrophage inflammatory protein 1 alpha (MIP-1alpha) in CSF and prevented the development of cortical injury [16,28]. During bacterial multiplication into the CSF, it released products that are highly immunogenic and can lead to an increased immune response of the host [10,16]. Whereas, in experimental pneumococcal meningitis model the animals presented, in the first twenty four hours, elevated levels of TNF-α and CINC-1 in the hippocampus and TNF-α, IL-1β, IL-6 and CINC-1 in frontal cortex [29]. Furthermore, the host inflammatory response can be exacerbated by the effects of bacteriolytic antibiotics [30]. The release of teichoic acids (TAs) and lipoteichoic acids (LTAs) from *S*. *pneumoniae* showed intense during exposure to ceftriaxone and meropenen [31]. Daptomycin is an antibacterial agent and nonbacteriolytic active against the main Gram-positive pathogens [11,32], including penicillin and cephalosporin resistant pneumococci [33]. Daptomycin was highly efficacious against penicillin-resistant and quinolone-resistant pneumococci [34], in addition, ceftriaxona with adjunctive daptomycin treatment attenuates brain damage and hearing loss more efficiently than rifampin in infant rats induced by pneumococcal meningitis [35]. When the microorganism is killed without lyses, it provides the advantage of reducing the release of bacterial molecules, such as, TAs, LTAs, peptidoglycan and bacterial DNA [36]. These effects could be an explanation for the observation that daptomycin prevented the development of cortical brain injury in experimental pneumococcal meningitis [16] and prevented memory impairment in our study. Previous studies demonstrated that 10 days after pneumococcal meningitis induction animals treated with ceftriaxone presented memory and learning deficits, anxiety-like and depressive-like behavior [37].

## Conclusions

The evidence of the present study suggests the potential alternative of the treatment with daptomycin in preventing learning and memory impairments caused by pneumococcal meningitis. Further investigations are necessary to provide support for evaluation of daptomycin as an alternative treatment for bacterial meningitis.

## Abbreviations

Cfu: colony-forming units; CINC-1: cytokine-induced neutrophil chemoattractant-1; CSF: cerebral spinal fluid; IL-1β: interleukin-1 betha; IL-6: interleukin-6; LTAs: lipoteichoic acids; S. pneumoniae: *Streptococcus pneumoniae*; TAs: teichoic acids; TNF-α: tumor necrosis factor-α

## Competing interest

The authors have no conflict of interest.

## Authors’ contributions

TB conceived of the study, and participated in its design and coordination. JCNG, JSG and GM have made substantial contributions to conception and design. CSC and JCR carried out the meningitis induction and acquisitons of data. CMC performed of the statistical analysis. JQ had given final approval of the version to be published. All authors read and approved the final manuscript.
